# Cell-Penetrating Doxorubicin Released from Elastin-Like Polypeptide Kills Doxorubicin-Resistant Cancer Cells in In Vitro Study

**DOI:** 10.3390/ijms22031126

**Published:** 2021-01-23

**Authors:** Jung Su Ryu, Felix Kratz, Drazen Raucher

**Affiliations:** 1 Department of Cell and Molecular Biology, University of Mississippi Medical Center, Jackson, MS 39213, USA; jryu@umc.edu; 2 CytRx Corporation, 79104 Freiburg, Germany; fkratz@cytrx.com

**Keywords:** drug delivery, tumor targeting, elastin-like polypeptide, cell penetrating peptide, matrix metalloproteinase, doxorubicin resistance

## Abstract

Elastin-like polypeptides (ELPs) undergo a characteristic phase transition in response to ambient temperature. Therefore, it has been be used as a thermosensitive vector for the delivery of chemotherapy agents since it can be used to target hyperthermic tumors. This novel strategy introduces unprecedented options for treating cancer with fewer concerns about side effects. In this study, the ELP system was further modified with an enzyme-cleavable linker in order to release drugs within tumors. This system consists of an ELP, a matrix metalloproteinase (MMP) substrate, a cell-penetrating peptide (CPP), and a 6-maleimidocaproyl amide derivative of doxorubicin (Dox). This strategy shows up to a 4-fold increase in cell penetration and results in more death in breast cancer cells compared to ELP-Dox. Even in doxorubicin-resistant cells (NCI/ADR and MES-SA/Dx5), ELP-released cell-penetrating doxorubicin demonstrated better membrane penetration, leading to at least twice the killing of resistant cells compared to ELP-Dox and free Dox. MMP-digested CPP-Dox showed better membrane penetration and induced more cancer cell death in vitro. This CPP-complexed Dox released from the ELP killed even Dox-resistant cells more efficiently than both free doxorubicin and non-cleaved ELP-CPP-Dox.

## 1. Introduction

It is acknowledged that current conventional chemotherapy is mostly comprised of cytotoxic drugs, which have a strong anticancer efficacy but cause collateral damage to non-tumor tissues. These unwanted side effects are usually a dose-limiting factor for chemotherapy and are a main reason for the unsatisfactory prognosis of the therapy. Many efforts have been made to resolve these problems, usually by attempting to raise the therapeutic index of the chemotherapy.

Curing cancer is certainly one of the greatest challenges of our time, and to confront it our knowledge of cancer has grown greatly over the last decades. In recent years, there has been a surge of new technologies for cancer treatment such as molecular targeted therapies (i.e., anti-tyrosine kinase and anti-HER2) [1], immunotherapies such as cancer vaccines or anti-PD1 [2,3], sophisticated radiation therapy [4], and advanced tumor-targeting technologies such as nanoparticles and antibody–drug conjugates. These technologies could make a big difference in the way we treat cancer, bringing us closer to being able to “cure” this disease. In particular, the nanosized drug delivery technologies have been significantly improved, and many of them are currently being used to solubilize the drugs, bypass immune surveillance, sensitize current therapies, and target tumor tissues [5,6]. A tumor-targeting technology delivers drugs specifically to tumor tissue so that the concentration of drugs in the tissue will increase compared to the concentration in normal tissue. This allows more opportunity for a drug to express its activity on tumor cells, resulting in the selective death of cancer cells with tolerable side effects.

An elastin-like polypeptide (ELP) is a thermo-responsive bio-polymeric carrier for targeted drug delivery. ELPs derived from tropoelastin consist of repeats of pentapeptides (mainly comprised of valine, proline, and glycine) [7,8]. The repeats of these hydrophobic amino acids permit an ELP to have a unique re-arrangement of molecules in response to the surrounding temperature, which is a thermoresponsive phase transition. At low temperature, an ELP remains a monomer and is soluble in solution; however, it co-acervates and precipitates in solution when the ambient temperature rises above its phase-transition temperature [9,10,11]. This co-acervation can also be reversed by decreasing the temperature of the ELP solution. Thus, this reversible phase transition of an ELP is mainly controlled by temperature, and the ELP is highlighted as a controllable carrier for the delivery of anticancer drugs in active tumor-targeting strategies.

Additionally, and ELP exploits an “enhanced permeability and retention (EPR) effect” and can progressively accumulate in tumor tissue due to the abnormal histological structures of the tumor. These unique properties turn the ELP itself into a drug carrier that can exploit both the EPR effect and tumor targeting using the hyperthermic technique [12]. Furthermore, the ELP has been modified by the addition of cell-penetrating peptides (CPPs) to allow enhanced cellular uptake, improved penetration of physiological barriers like the blood–brain barrier, and preferential intracellular distribution such as in the cytoplasm or the nucleus [13,14]. Many previous researchers have verified the potential of this polymer [15,16,17,18], and animal studies have demonstrated that ELPs are able to deliver a sufficient amount of a drug to the tumor area to produce significant tumor reduction efficacy in combination with the use of local hyperthermia. 

**Figure 1:**
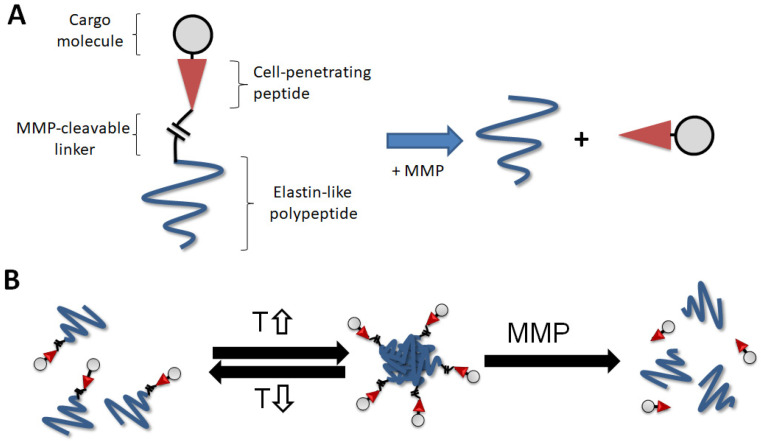
Elastin-like peptide (ELP) drug delivery system. (**A**) The proposed ELP system consists of an elastin-like polypeptide, matrix metalloproteinase (MMP)-cleavable linker, cell-penetrating peptide (CPP), and cargo molecules. The constructs are digested by the MMP, releasing the CPP cargo molecule. (**B**) The hypothetical model proposed in this system. The ELP constructs can form aggregates and release the CPP cargo molecule in hyperthermic tumors (T, temperature).

In this study, we further modified an ELP drug delivery system to release drugs in response to an enzyme that is abundant in tumor tissue (Figure 1). The suggested system is composed of an ELP; a matrix metalloproteinase (MMP) substrate, mmpL; a cell-penetrating peptide, CPP; and a 6-maleimidocaproyl amide derivative of doxorubicin, Dox (Figure 1A). We report the potential use of this strategy, an MMP-responsive ELP drug delivery system releasing CPP-Dox, to overcome Dox resistance by investigating the cellular uptake and anti-proliferation properties of the proposed system.

## 2. Results

### 2.1. Incubation of ELP-mmpL-CPP with MMP-2 Produces Cleaved CPP

**Figure 2:**
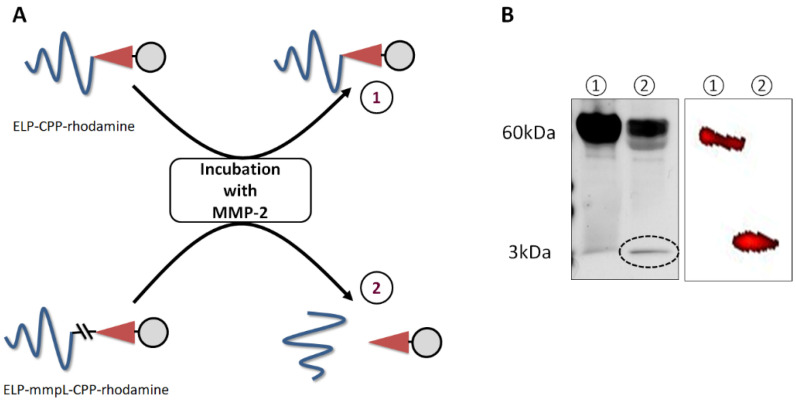
MMP-2 digestion of ELP-mmpL-CPP. (**A**) ELP-mmpL-CPP-rhodamine and ELP-CPP-rhodamine were incubated with MMP-2 for 4 h in ZnCl_2_ buffer (pH 7). (**B**) MMP-2 incubation of the constructs produced ELP-CPP-Dox (63 kDa, upper band) and CPP-rhodamine (3 kDa, lower band). Left panel: silver-stained gel; Right panel: fluorescence-scanned gel. Dox: 6-maleimidocaproyl amide derivative of doxorubicin; mmpL: MMP substrate.

Figure 2A depicts how ELP-mmpL-CPP would be cleaved by MMP, producing ELP (60 kDa) and cleaved CPP (Tat peptide, 3 kDa), while the other construct, ELP-CPP-Dox (63 kDa), would not be digested by MMP. This hypothesis was verified by the following experiments. MMP-2 was used for the digestion in this experiment since MMP-2 is involved in the degradation of extracellular matrices in tumors and is overexpressed in most tumors compared with normal tissues [19]. After incubation of each rhodamine (rho)-labeled construct (ELP-mmpL-CPP-rho and ELP-CPP-rho) with MMP-2, the reactant was run on SDS-PAGE and analyzed by both silver staining and fluorescence scanning (Figure 2B). Silver-stained gels revealed that MMP-digested ELP-mmpL-CPP-rho produced two bands (lane ② in the left panel); the upper one for ELP (60 kDa) and the other for cleaved CPP-rho (3 kDa), while ELP-CPP-rho digestion produced only one band (lane ① in the left panel) which represents undigested ELP-CPP-rho. However, when the gel was scanned for fluorescence, each reactant showed only one band. Since rhodamine was conjugated to the C-terminal of the CPP (Tat peptide), MMP digestion produced one single fluorescent band (CPP-rho, 3 kDa) without ELP (lane ② in the right panel), while a band of undigested ELP-CPP-rho fluoresced at around 63 kDa, as with the silver-stained gel (lane ① in the right panel).

### 2.2. MMP-2 Digestion Increases the Cellular Uptake of CPP -Rhodamine in Breast Cancer Cells

**Figure 3:**
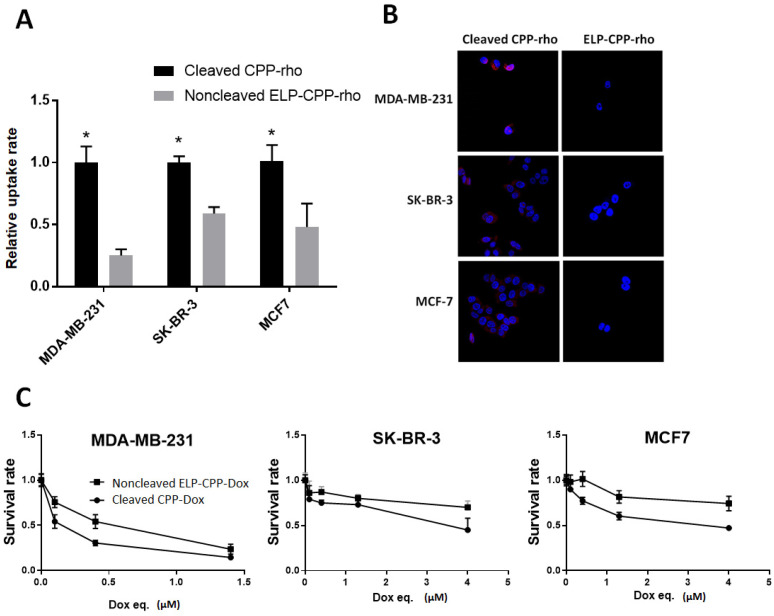
Cellular uptake rate of cleaved CPP-rhodamine in breast cancer cells. (**A**) Each cellular uptake rate was measured through flow cytometry (* *p* < 0.05). (**B**) Merged image of Dox (red) and DAPI (blue). (**C**) Cytotoxicity of cleaved CPP-Dox on breast cancer cells. Cells were treated with ELP-CPP-Dox and ELP-mmpL-CPP-Dox, both digested by MMP incubation.

MMP digestion will produce CPP-rhodamine (rho), which is smaller than the whole construct, ELP-mmpL-CPP-rho. This smaller size would facilitate its cellular uptake. Cells treated with MMP-digested ELP-mmpL-CPP-rho and ELP-CPP-rho, respectively, were analyzed for uptake ability via flow cytometry. In Figure 3A, cells treated with cleaved CPP-rho (from ELP-mmpL-CPP-rho) showed up to five times higher uptake rates than the ELP-CPP-rho-treated group in three cancer cell lines. This improved cellular uptake was also evident in observation with a fluorescence microscope (Figure 3B).

### 2.3. Cleaved CPP-Dox Kills Breast Cancer Cells More Efficiently than Non-Cleaved ELP-CPP

Rhodamine was replaced by doxorubicin to investigate whether improved uptake of cleaved CPP would contribute to cytotoxicity. Figure 3C compares the cytotoxicities of MMP-2-digested ELP-mmpL-CPP-Dox and ELP-CPP-Dox against three cancer cell lines. Improved cytotoxicity was observed in MMP-2-digested ELP-mmpL-CPP-Dox-treated cells than those treated with ELP-CPP-Dox. These results suggest that the MMP digestion of ELP-mmpL-CPP-Dox results in increased uptake of cargo molecules and facilitated the death of cancer cells by cleaved CPP-Dox.

### 2.4. Cleaved CPP-Dox Deposits in and Kills Dox-Resistant Cancer Cells

To investigate whether cleaved CPP-Dox is able to penetrate and kill even Dox-resistant cancer cells, comparisons of cytotoxicities and uptake rates of MMP-cleaved CPP-Dox were made between Dox-resistant cells (NCI/ADR, MES-SA/Dx5) and Dox-sensitive cells (MCF7, MES-SA).

**Figure 4:**
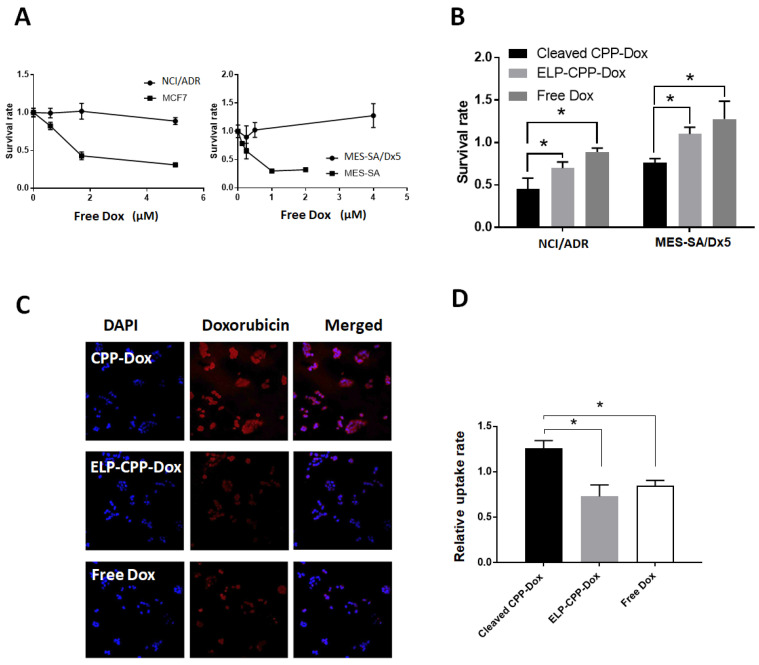
Cytotoxicity of CPP-Dox against Dox-resistant cancer cells. (**A**) Free Dox killed Dox-sensitive cancer cells (MCF7 and MES-SA), while it spared Dox-resistant NCI/ADR and MES-SA/Dx5. (**B**) Cytotoxicities of CPP-Dox and ELP-CPP-Dox in NCI/ADR and MES-SA/5DX at 4 µM Dox equivalence. (**C**) Confocal microscopic images show that CPP-Dox penetrated into NCI/ADR. (**D**) Flow cytometry, 60% increased uptake in CPP-Dox in comparison with ELP-CPP-Dox and free Dox. * *p* < 0.05.

Figure 4A shows the validated Dox resistance in NCI/ADR and MES-SA/Dx5, and cleaved CPP-Dox from ELP-mmpL-CPP-Dox showed more cell killing than ELP-CPP-Dox at 4 µM Dox equivalence. Confocal microscopic images of NCI/ADR cells show that cleaved CPP-Dox from ELP-mmpL-CPP-Dox was taken up by NCI/ADR more than the other constructs (i.e., free Dox and ELP-CPP-Dox; Figure 4B). This was also confirmed by flow cytometry (Figure 4C). The uptake rate of MMP-digested CPP-Dox in NCI/ADR was almost doubled compared with the uptake rates of free Dox and ELP-CPP-Dox. These results suggest that MMP-cleaved CPP-Dox can penetrate and kill even Dox-resistant cancer cells, probably with the help of a CPP (Tat peptide). One limitation of this experiment is that 4 µM of a doxorubicin-equivalent dose is the maximum concentration that can be reached from the current cleavage assay protocol; further optimization of the protocol may enable the generation of a higher concentration of each drug and calculation of IC50 to compare the cytotoxicity of each treatment.

### 2.5. MMP-Releasing HT-1080 Can Cleave ELP-mmpL-CPP-rho and Take up Cleaved CPP-rho

**Figure 5:**
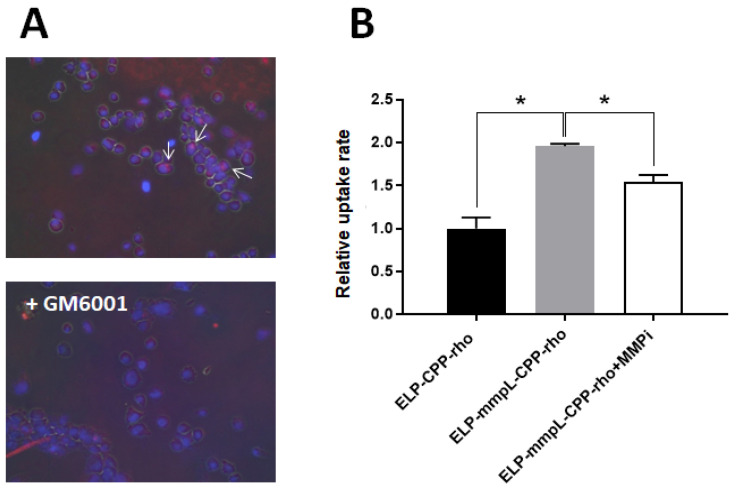
Cellular uptake rate of CPP-rhodamine in MMP-expressing HT-1080 cells. (**A**) Localization of CPP-rho (fluorescence microscopy, 20x) in cultured HT-1080 cells. The arrows indicate the CPP-rhodamine in the cells. (**B**) Flow cytometry showing increased uptake in cleaved CPP-rho in cells. * *p* < 0.05.

Given that an MMP-cleaved CPP-Dox can inhibit proliferation in Dox-resistant cancer cell lines, this ELP-mmpL-CPP-Dox system was further validated using HT-1080, a fibrosarcoma cancer cell producing endogenous MMP-2 and MMP-9. This experiment showed that the ELP-mmpL-CPP construct could also be digested by the endogenous MMP enzyme and release CPP cargo molecules. MMP-releasing HT-1080 cells were incubated with either ELP-mmpL-CPP-rho or ELP-CPP-rho for 4 h, and each group of treated cells was processed either for flow cytometry or fluorescence microscopy. In flow cytometry, cells incubated with the ELP-mmpL-CPP-rho group had twice the rhodamine signal of the ELP-CPP-rho group. However, this increased uptake was reversed by pretreatment with GM6001, an MMP catalytic inhibitor (Figure 5A). This finding was further confirmed by fluorescence microscopy, with the rhodamine particles being found in the nucleus of HT-1080 cells treated with ELP-mmpL-CPP-rho (Figure 5B). Uptake of these particles, as in the flow cytometry experiment, was also abolished by GM6001 pretreatment. GM6001 prevents MMP digestion, and undigested ELP-mmpL-CPP-rho was likely washed off the cells during the rinsing step. These results indicate that ELP-mmpL-CPP-rho was digested by intrinsic MMP released from HT-1080 cells, and that the resultant cleaved CPP-rho penetrated the HT-1080 cells.

## 3. Discussion

Our tumor-targeted drug delivery system using an ELP delivers anticancer cargo molecules specifically to the tumor site by exploiting the enhanced permeability and retention (EPR) effect along with the active thermal targeting approach [15,16,17,20]. This thereby increases the relative concentration of the cargo drugs in tumors and improves the therapeutic index of the drugs, alleviating unacceptable toxicity to the patients [12,21]. A striking example of this targeting can be found in previous studies [20], in which fluorescently labeled CPP-ELPs were administered into S2013 tumor-bearing mice. One group of animals received hyperthermic treatment with infrared (IR) lasers on tumors immediately after injection of CPP-ELP so that the temperature in the tumor core reached 42 °C, while the other group was exempt from hyperthermic treatment. This study demonstrated that the IR heating of tumors created 2–3 times greater tumor accumulation of CPP-ELP as well as the penetration of this protein into the tumor tissues. Given the thermoresponsive behavior of the ELP, the aggregation of CPP-ELP in hyperthermic tumors resulted in an increase of the construct’s concentration in the tumors. These results strongly suggest that the ELP preferentially accumulated in tumors in response to local hyperthermia.

We further improved the ELP system in this study to release payloads in response to additional external stimuli—that is, matrix metalloproteinases. This novel system is comprised of four components: an ELP, an MMP-2-cleavable linker, CPP (Tat peptide), and doxorubicin as a payload. The linker is a substrate of MMP-2, designed to be cleaved by MMP-2 so that the ELP system can eventually release a complex of the payload (doxorubicin) and a CPP (CPP-Dox) in tumor tissues. The involvement of MMPs, which are zinc-containing endopeptidases, in cancer biology has been extensively discussed in a variety of publications [22,23,24,25]. Especially, increased expression and activity of MMP-2 and MMP-9 subtypes in tumors are known to be related to the degradation of basement membranes—an essential step in tumor invasion and in enhancing angiogenesis. For example, Tutton et al. reported that MMP-2 expression was significantly increased in colorectal cancer tissues compared to matched normal colon tissue as measured by ELISA [26]. High levels of MMPs in tumors will facilitate the release of CPP-Dox out of the renovated ELP complex and provide an additional, secondary tumor-targeting opportunity compared to the previous ELP delivery system. This system thus becomes a triple-targeting strategy when used along with the EPR effect and local hyperthermia. Specifically, this cleavable ELP construct still contains the ELP molecule until it is digested by MMPs at tumor tissues. This late cleavage process will allow CPP-Dox to benefit from ELP by EPR, and from the thermo-targeting. ELP is expected to allow the proposed construct to be initially targeted to the tumor site by the local application of mild heat. Then, ELP-mmpL-CPP-Dox will be fully digested by MMP to release CPP-Dox (Figure 1B), followed by improved cellular uptake by cancer cells and increased cancer-cell death.

This MMP-cleavable system displays a couple of other advantages in delivering chemotherapeutic molecules. First, when the MMP-cleavable ELP-CPP-drug is digested by MMP in tumor tissue, small fragments (CPP-Dox) will be produced. Since the molecular weight of the released CPP-Dox (<3 kDa) is one-twentieth that of the parental ELP construct (60 kDa), it will quickly infiltrate into adjacent tumor cells, as can be seen in other studies [27,28]. This hypothesis was examined by cell-uptake assays in this study. Cells treated with CPP-rho, which is a digested product from ELP-mmpL-CPP-rho, showed more rhodamine uptake than the cells treated with undigested ELP-CPP-rho. It is also demonstrated in Figure 5 that the ELP-mmpL-CPP-rho could be digested by endogenous MMPs and taken up by HT-1080 cells. Reversal of this uptake by GM6001 (an MMP inhibitor) indicates that the cell uptake of rhodamine by HT-1080 relies on the catalytic activity of MMP. This increased uptake was reflected in the enhanced cytotoxicity of MMP-cleaved CPP-Dox in breast cancer cells. After MMP-2 digestion, ELP-mmpL-CPP-Dox killed more cancer cells than did ELP-CPP-Dox (Figure 3C).

A second advantage of this system is that the released CPP-Dox still takes advantage of the abilities of the CPP to facilitate uptake by the cells and to penetrate physiological barriers like the blood–brain barrier. More importantly, there is an increasing number of studies reporting the role of CPPs in overcoming the multidrug resistance (MDR) of cancer cells, which has been one of main hurdles that doxorubicin has faced. The use of doxorubicin, one of the most effective chemotherapy agents since the 1960s, has been compromised by the development of MDR in patients [29,30]. MDR involves increased efflux, decreased uptake, and enzymatic drug metabolism (e.g., glutathione S-transferase) of chemotherapeutic drugs such as doxorubicin [31]. An elevated expression of active drug transporters in cancer cells is known to be a major resistance mechanism [32]. The coupling of chemotherapeutic drugs to peptides such as CPPs have been suggested as the solution for these problems, since this strategy may alter the cellular uptake pathway and circumvent ABC-transporter-mediated drug efflux, allowing drugs to accumulate at high concentrations in drug-resistant cells, leading to an improved therapeutic index and fewer adverse effects [28,33,34,35]. Specifically, CPP-Dox developed by Liang et al. [35] showed a 59% uptake rate in Dox-resistant MCF7 cells, while 90% of free Dox was lost during cell penetration, leading to a considerable improvement in the IC_50_ of doxorubicin. In line with these reports, our current study also demonstrates that cleaved CPP-Dox showed greater cellular uptake by Dox-resistant NCI/ADR in comparison with free Dox and ELP-CPP-Dox (Figure 4C). Like Dox-sensitive breast cancer cells, this increased uptake also led to an enhanced cytotoxicity of cleaved CPP-Dox against NCI/ADR and MES-SA/Dx5 (Figure 4B).

In summary, the modified ELP-CPP-Dox was cleaved by incubation with an intrinsic or extrinsic MMP enzyme. MMP digestion produced CPP-Dox (or rhodamine), which showed better membrane penetration and induced more cancer cell death *in vitro*. This CPP-complexed Dox released from an ELP killed even Dox-resistant cells more efficiently than both free doxorubicin and non-cleaved ELP-CPP-Dox. This pilot study emphasizes the extra functionalities of the ELP drug delivery system. The novelty of this study is improvement in the drug delivery efficiency of ELP and demonstration of ELPs’ potential in multiple-targeting strategies. 

## 4. Materials and Methods

### 4.1. Design of Construct and Protein Preparation

The ELP used in this study consists of 150 repeats of VPGXG with guest residues (amino acid at position X) of Val, Gly, and Ala in a 5:3:2 ratio. ELP coding sequences were modified by the addition of the “Tat” cell-penetrating peptide sequence (YGRKKRRQRRR), an MMP-cleavable sequence (PLGALG), and three Gly-Gly-Cys residues to the C-terminus of the ELP for the conjugation with Dox (Table 1). For an MMP-uncleavable control, six Gly residues were used instead of the MMP-cleavable sequence. All constructs were expressed in the *Escherichia coli* strain BLR (DE3) using pET 25b as an expression vector, and were purified by repeated inverse transition cycling.

### 4.2. Conjugation of ELP Constructs with Doxorubicin or Fluorescent Probes

Protein (100 µM) in PBS was reduced with 1 mM of tris-(2-carboxyethyl) phosphine (TCEP, Molecular probes) for 30 min at room temperature. Conjugation with 200 µM of the 6-maleimidocaproyl amide derivative of doxorubicin or tetramethylrhodamine-5-iodoacetamide dihydroiodide (molecular probes) was followed by incubation at 4 °C overnight. Conjugated peptides were purified by inverse transition purification as described previously [9], and the concentration and the labeling efficiency were assessed by UV–visible spectrometry (UV-1600, Shimadzu). Concentrations of labeled ELP polypeptides were determined using the following equations:

for Dox conjugation

$$\mathrm{protein},\;M\;=\;\frac{\mathrm{Abs}\;280\mathrm{nm}-(0.71\;\times \;\mathrm{Abs}\;495\mathrm{nm})}{6890M-1{\mathrm{cm}}^{-1}}$$

for tetra-methyl-rhodamine conjugation

$$\mathrm{protein},\;M=\frac{\mathrm{Abs}\;280\mathrm{nm}-(0.17\;\times \;\mathrm{Abs}\;541\mathrm{nm})}{5690M-1{\mathrm{cm}}^{-1}}$$

### 4.3. Cell Culture

MDA-MB-231, MCF7, NCI/ADR, MES-SA, MES-SA/Dx5, and SKBR3 cell lines were obtained from ATCC. HT-1080 was a generous gift from Dr. Michael Herbert of the University of Mississippi Medical Center. All cell lines were grown and maintained at 37 °C, 5% CO_2_ in Dulbecco’s Modified Eagle’s Medium with 10% fetal bovine serum.

### 4.4. Cleavage Assays

Recombinant human pro-MMP-2 (Enzo life science) was activated with 2.5 mM 4-aminophenylmercuric acetate at 37 °C for 2 h. Then, 1 µg of each ELP construct was incubated with the pre-activated MMP-2 (10 pmol) for 4 h in a reaction buffer (50 mM Tris, 200 mM NaCl, 10 mM CaCl_2_, and 10 mM ZnCl_2_, pH 7.5). After the reactions, the samples were loaded and separated on an SDS-PAGE gel, and each peptide’s cleavage pattern was confirmed by silver staining and by scanning the fluorescence of the gel with the IVIS Live Animal Imager (Caliper Life Sciences).

### 4.5. Flow Cytometry Analysis of Cellular Uptake

Cells were incubated with each treatment for 2 h at 37 °C, rinsed with PBS, and collected by trypsinization. Intracellular fluorescence was measured using a Gallios Flow Cytometer (Beckman Coulter) after trypan-blue quenching as described previously [36]. Forward- versus side-scatter gating was used to remove cell debris from the analysis, and the mean cellular fluorescence intensity was recorded. The mean cellular fluorescence was corrected for differences in labeling efficiencies among polypeptides, and the results shown are an average of at least 3 experiments with bars representing the standard error of the mean (SEM).

### 4.6. Cytotoxicity Test

Cells were plated in a 96-well plate and treated with a range of concentrations of each treatment for 24 h at 37 °C. After further incubation with fresh media for 48 h, cell viability was assessed using the MTT assay (Sigma). Briefly, a 0.5 mg/mL solution of thiazolyl blue tetrazolium bromide dissolved in PBS was added to each well and the plates were incubated for 4 h at 37 °C. Formazan formed by mitochondrial reduction was dissolved in 100 µL of DMSO, and its absorbance was read at 570 nm. The survival rate of each group was calculated in comparison to a vehicle-treated control group.

### 4.7. Confocal Microscopy

Cells (10^3^ cells/chamber) were plated in 2-well Lab-Tek CC2 chamber slides (Nunc). After 24 h incubation at 37 °C, the cells were treated with each treatment for two hours at 37 °C. The cells were washed three times with PBS, fixed with cold 4% paraformaldehyde, and stained with DAPI (molecular probe) for 10 min at room temperature to visualize the nucleus. Distribution of each molecule was investigated by laser scanning confocal microscopy with a 60x oil immersion objective (Leica).

## Figures and Tables

**Table 1:** Construct sequences.

Construct	Sequence
MMP-cleavable ELP-mmpL-CPP	ELP-(PLGALG)-CPP-(GGC)3
MMP-uncleavable ELP-CPP	ELP-GGGGGG-CPP-(GGC)3

## Data Availability

The data that support the findings of this study are available from the corresponding author upon reasonable request.

## Abbreviations

ELP	Elastin-like polypeptide
MMP	Matrix metalloproteinase
CPP	Cell-penetrating peptide
Dox	Doxorubicin
MDR	Multidrug resistance
EPR	Enhanced permeability and retention
Rho	Rhodamine
mmpL	MMP-cleavable linker
